# Therapeutic Target Analysis and Molecular Mechanism of Melatonin - Treated Leptin Resistance Induced Obesity: A Systematic Study of Network Pharmacology

**DOI:** 10.3389/fendo.2022.927576

**Published:** 2022-07-22

**Authors:** Vennila Suriyagandhi, Vasanthi Nachiappan

**Affiliations:** Biomembrane Lab, Department of Biochemistry, School of Life Sciences, Bharathidasan University, Tamilnadu, India

**Keywords:** melatonin, obesity, leptin resistance, bioinformatic analysis, network topological analysis

## Abstract

**Background:**

Obesity is a medical problem with an increased risk for other metabolic disorders like diabetes, heart problem, arthritis, etc. Leptin is an adipose tissue-derived hormone responsible for food intake, energy expenditure, etc., and leptin resistance is one of the significant causes of obesity. Excess leptin secretion by poor diet habits and impaired hypothalamic leptin signaling leads to LR. Melatonin a sleep hormone; also possess antioxidant and anti-inflammatory properties. The melatonin can attenuate the complications of obesity by regulating its targets towards LR induced obesity.

**Aim:**

The aim of this study includes molecular pathway and network analysis by using a systems pharmacology approach to identify a potential therapeutic mechanism of melatonin on leptin resistance-induced obesity.

**Methods:**

The bioinformatic methods are used to find therapeutic targets of melatonin in the treatment of leptin resistance-induced obesity. It includes target gene identification using public databases, Gene ontology, and KEGG pathway enrichment by ‘ClusterProfiler’ using the R language, network analysis by Cytoscape, and molecular Docking by Autodock.

**Results:**

We obtained the common top 33 potential therapeutic targets of melatonin and LR-induced obesity from the total melatonin targets 254 and common LR obesity targets 212 using the data screening method. They are involved in biological processes related to sleep and obesity, including the cellular response to external stimulus, chemical stress, and autophagy. From a total of 180 enriched pathways, we took the top ten pathways for further analysis, including lipid and atherosclerosis, endocrine, and AGE-RAGE signaling pathway in diabetic complications. The top 10 pathways interacted with the common 33 genes and created two functional modules. Using Cytoscape network analysis, the top ten hub genes (TP53, AKT1, MAPK3, PTGS2, TNF, IL6, MAPK1, ERBB2, IL1B, MTOR) were identified by the MCC algorithm of the CytoHubba plugin. From a wide range of pathway classes, melatonin can reduce LR-induced obesity risks by regulating the major six classes. It includes signal transduction, endocrine system, endocrine and metabolic disease, environmental adaptation, drug resistance antineoplastic, and cardiovascular disease.

**Conclusion:**

The pharmacological mechanism of action in this study shows the ten therapeutic targets of melatonin in LR-induced obesity.

## Introduction

According to WHO, obesity is a global health challenge in the current century. It recognized obesity as an epidemic with increased BMI. Around 30% of the global population is affected by obesity due to poor eating habits and sedentary lifestyles (1). A study by Institute for Health Metrics and Evaluation (IHME) with the Global Burden of Disease 2019 estimated that the worldwide health loss has been increased (50%) compared to 1990 (10.4%). The interaction of COVID-19 with rise in chronic illness leads to increased deaths caused by pandemic (2). The major cause of death particularly 1 in 5 deaths are related to high blood pressure (11 million). Next leading causes of deaths are high blood sugar (6.5 million) and high cholesterol (4.4 million) (3). Obesity with high BMI led to major diseases like T2DM and cardiovascular disease (4). Obesity is an imbalance of food intake, and energy expenditure leads to adipose tissue enlargement (5). High fat, high carbohydrate consumption, and altered environmental factors play a significant role in developing obesity, leading to severe dysfunction of white adipose tissue (6, 7).

Many of the co−morbidities of obesity are related to chronic inflammation and include heart diseases, non-alcoholic fatty liver disease, steatohepatitis, cancer, T2DM, and neurodegenerative diseases (8). In a study by Boi et al. (9), diet-induced obesity altered immune and metabolic profiles. In proportion to the amount of stored fat in adipocytes, our body secretes a hormone called leptin. Leptin is a peptide hormone that controls food intake and energy expenditure by sending signals to the brain, especially the hypothalamus (10), to reduce obesity and its complications. However, significant obese populations are found to have high circulating leptin levels and are resistant to leptin treatment and are leptin resistant (11). To exert its function in the hypothalamus, leptin must cross the blood-brain barrier (BBB) through some specific transporters. Decreased leptin sensitivity in obese subjects may be due to reduced BBB permeability by saturation of its transporters (11). In addition, blockage of leptin signaling by continuous overstimulation of the leptin receptor in the hypothalamus leads to leptin resistance (12).

Ghanemi et al. reported that regeneration of the body is known as the maintenance of a healthy body by normal growth and development. Exercise, diet, and sleep are vital factors for body regeneration (13). Sleep deprivation is one of the major risk factors for developing obesity (14), and evidence shows that chronic sleep deprivation leads to body weight gain (15). Serotonin, melatonin and histamine are the major indoleamines that play an important role in health and disease. A similar bioinformatic study by Liu et al, revealed that the serotonergic neurons are involved in the etiology and therapy genetics of anxiety disorders (16). Serotonin is the precursor for melatonin production and is involved in satiety and feeding behaviors. It also regulates insulin in pancreatic β cells and leptin from adipocytes. At the hypothalamic level melatonin interacts particularly with 5-HT_6_ receptors in association with α-MSH, orexin and leptin (peripheral satiety signal) (17).

The pineal gland secretes the sleep hormone melatonin in the night and regulates the circadian rhythm and body temperature based on 24 h light/dark cycles (18). Melatonin is an effective antioxidant and possesses free radical scavenging and anti-inflammatory activities. A bioinformatic study by Yang et al. explained the possible pharmacological mechanisms in treating diminished ovarian reserve by melatonin (19). Melatonin activates antioxidant enzymes at the mRNA level and increases anti-inflammatory activity and immune function (20). It reduces the risk of oxidative stress and cancer and is also responsible for modulating the neuroendocrine reproductive axis (21, 22). In addition, melatonin plays an essential role in maintaining the proper functioning of energy metabolism, including lipid and carbohydrate metabolism (23). Supplementation of melatonin in several experimental models decreased adipose tissue levels and the body weight (24). Supplementation of melatonin in experimental young animals with intact melatonin secretion reduced long-term body weight gain. The anti-obesogenic effect of melatonin is observed with diet-induced obesity (7).

Melatonin contributes to the regular expression and leptin secretion pattern (25), and the lack of melatonin signaling is responsible for the development of leptin resistance (26). Melatonin supplementation for 12 weeks in middle-aged rats reduced intra-abdominal adiposity, body weight gain, and plasma leptin level (27). Treatment of melatonin in diet-induced obese mice reported reduced leptin mRNA expression and inflammatory cytokines in white adipose tissue (28). Administration of melatonin to a pineal gland removed animal improved leptin sensitivity and activated leptin signaling pathway (29).

**Figure 1:**
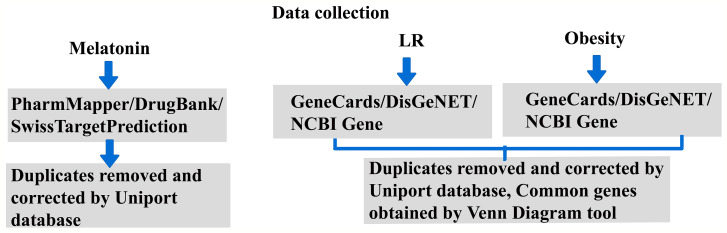
Research Work outline.

Our previous report (30) explained the existing knowledge gap and future directions for LR-induced obesity by treatment with melatonin. The role of the regulatory pathway and signaling mechanism will give a new perspective on melatonin treatment for obesity. Hence, this revealed the therapeutic mechanism of melatonin by potential predictive targets of LR-induced obesity using a systematic network pharmacological -based analysis. **Figure 1** shows the work outline of the study.

## Materials and Methods

### Finding Melatonin Targets

The Simplified Molecular-Input Line-Entry System (SMILES) (31) and melatonin 3D structure were obtained from the PubChem database (https://pubchem.ncbi.nlm.nih.gov/). The possible targets for melatonin were spotted using the PharmMapper Server (http://lilab-ecust.cn/pharmmapper/index.html) (32), and SMILES information was used for the SwissTargetPrediction database (http://swisstargetprediction.ch/) (33). The DrugBank database (https://go.drugbank.com/) (34) and Comparative Toxicogenomics Database (https://ctdbase.org/) (35) identifies the drug-gene and chemical-gene interactions. After removing duplicates, the collected target names and IDs were amended using the UniProt database (https://www.uniprot.org/).

### Targets of Leptin Resistance and Obesity

The targets of obesity and leptin resistance were separately retrieved from the databases GeneCards (https://www.genecards.org/) with the threshold of relevance score set at a minimum of 10, DisGeNET Score was set at a minimum of 0.1 (https://www.disgenet.org/) (36) and NCBI Gene (https://www.ncbi.nlm.nih.gov/gene) with the keywords leptin resistance and obesity. Duplicates were removed with the aid of Venn Diagram tool (https://bioinformatics.psb.ugent.be/webtools/Venn/); common intersecting genes of leptin resistance and obesity were obtained (37). We found 212 common targets and used them for further analysis.

### PPI Network Construction and Identification of Hub Genes

The STRING (Search Tool for the Retrieval of Interacting Genes) database aids to anticipate protein-protein interactions. STRING database version 11. 5 was used to predict the PPI network of gene lists with a high confidence interaction score of 0.700 (38). The predicted PPI network was visualized by Cytoscape (v3.9.0) and using Maximal Clique Centrality (MCC) topological analysis, the foremost 10 core genes were recognized by Cytoscape plugin CytoHubba (39). For network construction, we used Cytoscape software (1) melatonin with its targets, (2) LR with Obesity and its PPI interaction, (3) PPI interaction of common targets of melatonin and LR with Obesity, (4) functional modules of top 10 network using clustermaker2 Glay (Community Cluster) algorithm, and (5) subnetworks of therapeutic targets enriched in different pathway classes (19).

### The GO Enrichment and KEGG Pathway Analysis

The ClusterProfiler package (A universal enrichment tool for interpreting omics data) of the R program (4.1.2) used for the Gene Ontology and KEGG pathway enrichment analysis (40) of the common 33 targets of melatonin and LR with Obesity. The GO enrichment analysis was done by enrichGO and KEGG pathway enrichment analysis was done by enrichKEGG options of ClusterProfiler. plots were developed using ggplot2 to create GO (Chord diagram), enrichKEGG (Dot plot & Sankey plot) and pathway class graph. We used KEGG pathway database (https://www.kegg.jp/) for the pathway class analysis of the top 33 targets.

### Molecular Docking

#### Protein Preparation for Docking

The RCSB Protein Data Bank (https://www.rcsb.org/) provided the target proteins 3D crystal structures in PDB format. The CASTp – Computed Atlas of Surface Topography of proteins (http://sts.bioe.uic.edu/castp/index.html?4jii) database used to predict the active site amino acids and binding pocket details of target proteins. We used Autodock tools (1.5.7) to add charges and remove water from the protein molecule, and the PDBQT format of proteins was prepared (41).

#### Ligand Preparation for Docking

The 3D SDF structure of melatonin downloaded from PubChem database was converted into PDB format with the help of Open Babel software (3.1.1) (42). The.pdbqt format of melatonin was constructed using Autodock tools (1.5.7)

#### Docking and Visualization

Docking of the target protein with melatonin and its binding affinity was calculated using Autodock (4.2) software. DS visualizer software used to visualize the binding of melatonin with target proteins with best binding pose.

## Results

### Melatonin Target Network

**Figure 2:**
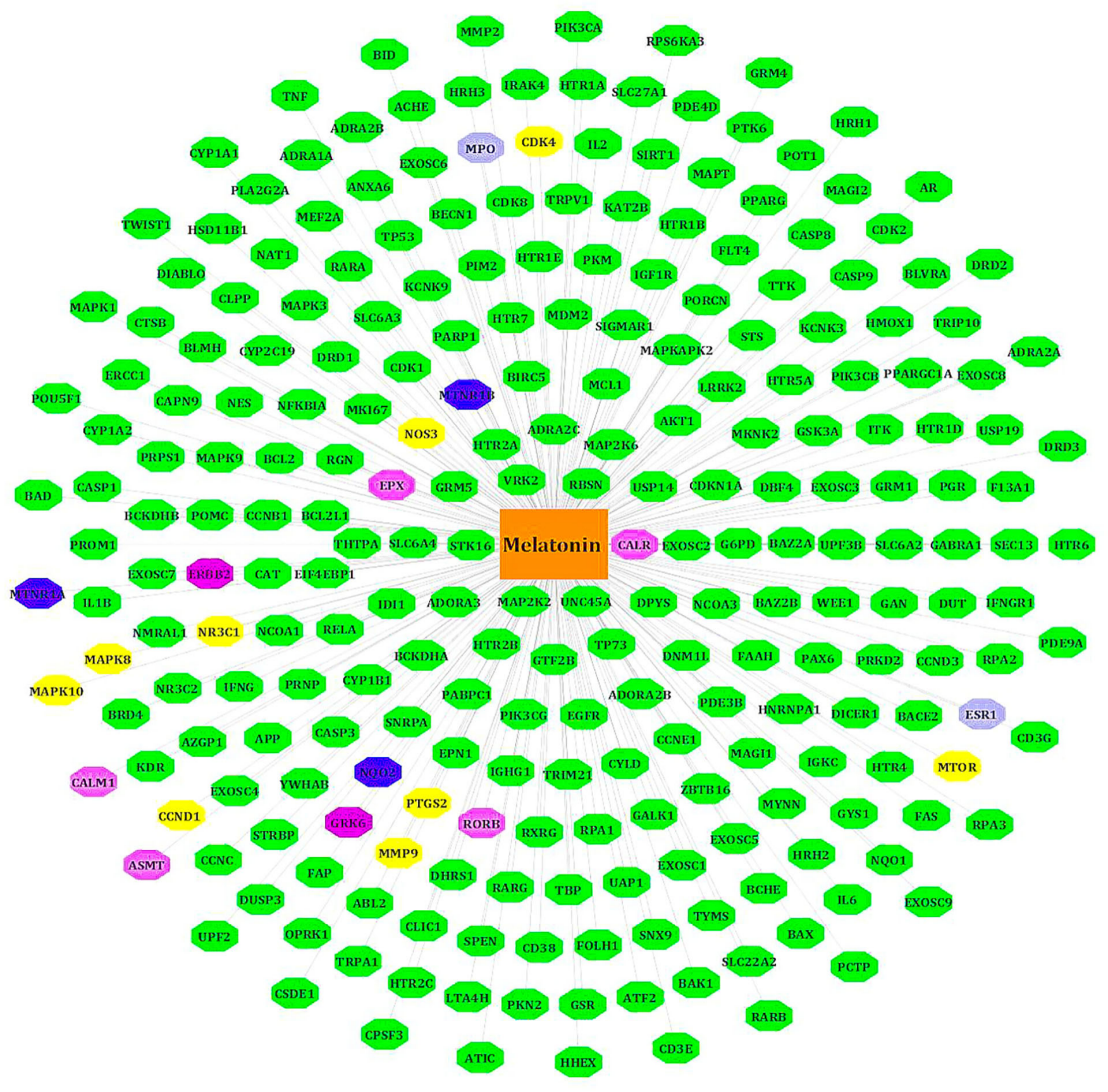
Target genes of melatonin. All known targets of melatonin are in green nodes. The Blue nodes -intersection of Swiss target prediction/CTD/DrugBank. Purple nodes - Swiss target prediction/Pharm Mapper. Yellow nodes - Swiss target prediction/CTD. Gray nodes - DrugBank/Swiss target prediction or CTD. Pink nodes – DrugBank.

We retrieved 254 melatonin targets from SwissTargetPrediction, PharmMapper, DrugBank, and CTD (Comparative Toxicogenomics Database). Using Cytoscape 3.9.0, duplicates were removed, and we constructed the melatonin target network. The intersecting targets among databases are colored differently (**Figure 2**).

### LR-Induced Obesity Targets and PPI Network

**Figure 3:**
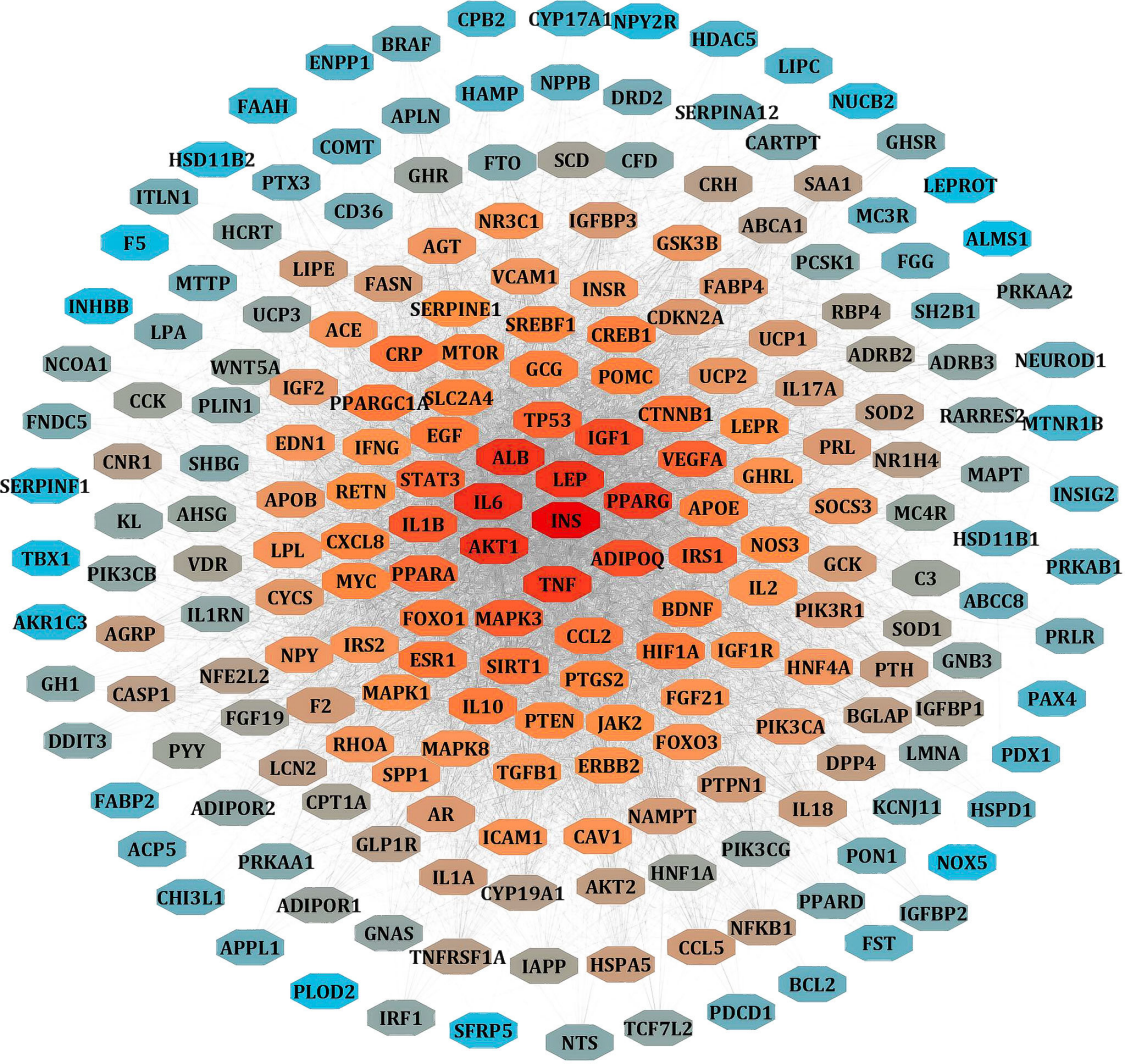
PPI network of LR-induced obesity. The node color red to blue represents the descending order of degree values.

We separately collected the obesity and leptin resistance targets from databases like DisGeNET, GeneCards, and NCBI Gene databases. We obtained a total of 212 common intersecting targets of LR and Obesity. These were the key targets for developing LR-induced obesity. Using analyze network option of Cytoscape in string interaction network [by an above-average of Degree Centrality (96.69811321), Betweenness Centrality (0.003931137), and Closeness Centrality (0.556192402)], a total of 48 significant targets common for LR and Obesity was obtained (**Figure 3**).

### PPI Network of Common Potential Melatonin and LR-Induced Obesity Targets

**Figure 4:**
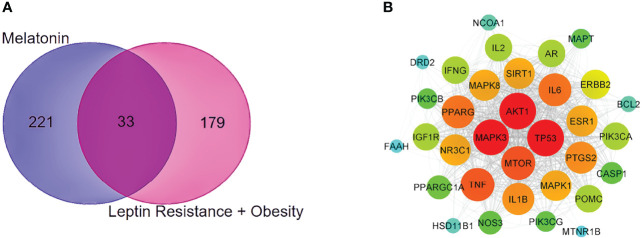
PPI network and Venn diagram of melatonin remedial targets in LR induced obesity. **(A)** The common targets of melatonin and LR induced obesity are depicted in the Venn diagram intersection. **(B)** PPI network of common remedial targets. The node sizes from large to small and color from red to cyan indicate the degree values in descending order.

With the help of the Venn Diagram tool (https://bioinformatics.psb.ugent.be/webtools/Venn/), the 33 potential targets common for melatonin and LR-induced obesity were obtained (**Figure 4A**). The network was constructed using Cytoscape and MCC score using the cytoHubba plugin the top 10 hub targets (TP53, AKT1, MAPK3, PTGS2, TNF, IL6, MAPK1, ERBB2, IL1B, MTOR) were obtained (**Figure 4B**). These ten targets might play an essential role in the melatonin treatment for LR-induced Obesity.

### GO Analysis

**Figure 5:**
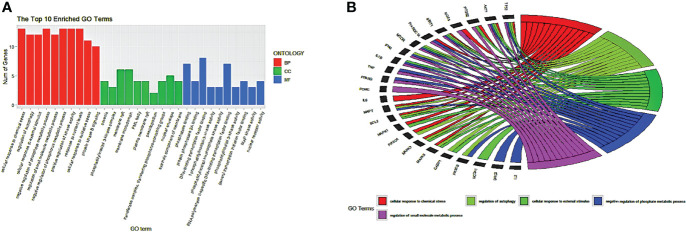
GO enrichment analysis and top five biological processes. **(A)** GO enrichment analysis. Top 10 significantly enriched terms of each part. BP, biological process; CC, cellular component; MF, molecular function. **(B)** The top 5/10 enriched biological processes.

Using the ClusterProfiler package of R language 4.1.2, the common 33 targets were analyzed, and we selected the top 10 GO terms based on p-value and number of counts. As shown in **Figure 5A**, we visualized the GO results using the ggplot2 package of R. The top 5 biological processes (BP) were selected based on p-value and number of counts and plotted using the R Chord Diagram (**Figure 5B**). The top 5 enriched BP were directly involved with melatonin treatment in LR-induced Obesity; the impact of cellular response to chemical stress, external stimulus, autophagy, negative regulation of phosphate metabolic process, regulation of small molecule metabolic process. Nine out of ten hub genes were enriched in top 5 BP (TP53, AKT1, MAPK3, PTGS2, TNF, IL6, MAPK1, IL1B, MTOR).

### KEGG Pathway Enrichment Analysis

**Figure 6:**
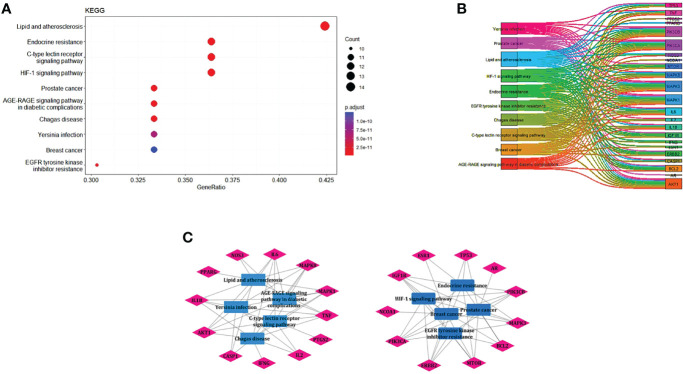
The KEGG pathway enrichment analysis of the common 33 therapeutic targets. **(A)** Top 10 significantly enriched pathways. The dot sizes from large to small and colors from red to blue indicate count of genes and p-*value* in descending order. **(B)** Genes involved in the top 10 enriched pathways. **(C)** Community cluster module analysis of the target pathway network. Blue nodes are the pathways, and pink nodes are the genes involved in each module.

**Figure 7:**
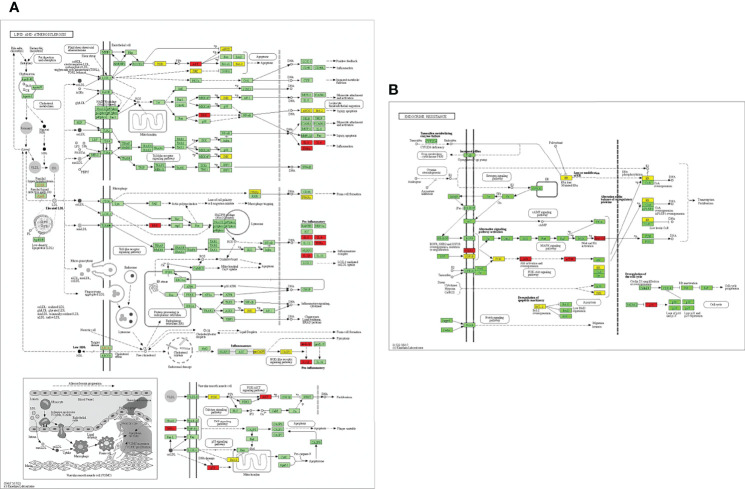
Distribution of potential targets on vital pathways: The nodes in red represent the hub genes; nodes in yellow represent common 33 target genes and nodes in green represents the other targets of pathways [**(A)** Lipid and atherosclerosis and **(B)** endocrine resistance].

**Figure 8:**
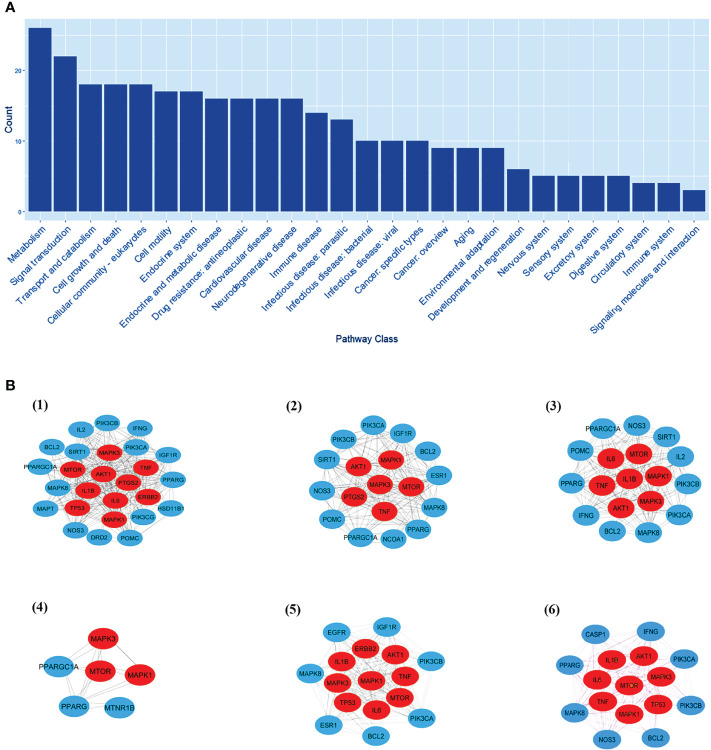
The KEGG pathway class analysis. **(A)** The KEGG pathway class analysis of top 33 targets. (**B**, 1-6) Subnetworks of major pathway classes.

EnrichKEGG pathway analysis for common 33 targets were done using ClusterProfiler R 4.1.2. We obtained a total of 180 pathways (p-value <0.05), took the top 10 pathways, and performed the dot-plot (**Figure 6A**). The genes enriched in individual pathways were constructed (**Figure 6B**). The top 10 pathways were decomposed using the Cytoscape Glay (community cluster) algorithm of clustermaker2 to understand the mechanism of melatonin in treating LR-induced Obesity. The top 10 pathways were divided into two functional modules (**Figure 6C**). Module 1 consists of five pathways, including lipid and atherosclerosis (hsa05417), Yersinia infection (hsa05135), signaling pathway of C-type lectin receptor (hsa04625), Chagas disease (hsa05142), signaling pathway (AGE-RAGE) in diabetic complications (hsa04933). Module 2 contained five pathways, including endocrine resistance (hsa01522), HIF-1 signaling pathway (hsa04066), Prostate cancer (hsa05215), EGFR tyrosine kinase inhibitor resistance (hsa01521), Breast cancer (hsa05224). The significantly enriched lipid and atherosclerosis (hsa05417) pathway and endocrine resistance (hsa01522) pathway were further analyzed by the KEGG pathway (**Figure 7** and **Table 1**). A total of 180 pathways were classified in different biological systems using the KEGG database. The related melatonin and LR-induced obesity-related six pathways were further divided into six subnetworks (**Figures 8A, B**).

**Table 1:** The KEGG pathway results.

Pathway class	Pathway ID	Pathway	Count	Total genes	p-*value*
Immune system	hsa04625	C-type lectin receptor signaling pathway	12	151	7.00E-15
Drug resistance: antineoplastic	hsa01522	Endocrine resistance	12	159	1.31E-14
Signal transduction	hsa04066	HIF-1 signaling pathway	12	182	6.73E-14
Cancer: specific types	hsa05215	Prostate cancer	11	148	2.40E-13
Cardiovascular disease	hsa05417	Lipid and atherosclerosis	14	346	2.94E-13
Infectious disease: parasitic	hsa05142	Chagas disease	11	163	6.99E-13
Endocrine and metabolic disease	hsa04933	AGE-RAGE signaling pathway in diabetic complications	11	166	8.54E-13
Drug resistance: antineoplastic	hsa01521	EGFR tyrosine kinase inhibitor resistance	10	119	1.01E-12
Infectious disease: bacterial	hsa05135	Yersinia infection	11	210	1.12E-11
Cancer: specific types	hsa05224	Breast cancer	11	217	1.61E-11

The top 10 enriched KEGG pathway classes of common 33 genes.

### Molecular Docking

For molecular docking analysis with melatonin, ten hub genes were chosen. Using CastP, we predicted the active site parameters of each target. Conventionally the target binding with lower affinity means stronger binding ability with melatonin. The strongly bound targets with melatonin were chosen for analysis.

**Figure 9:**
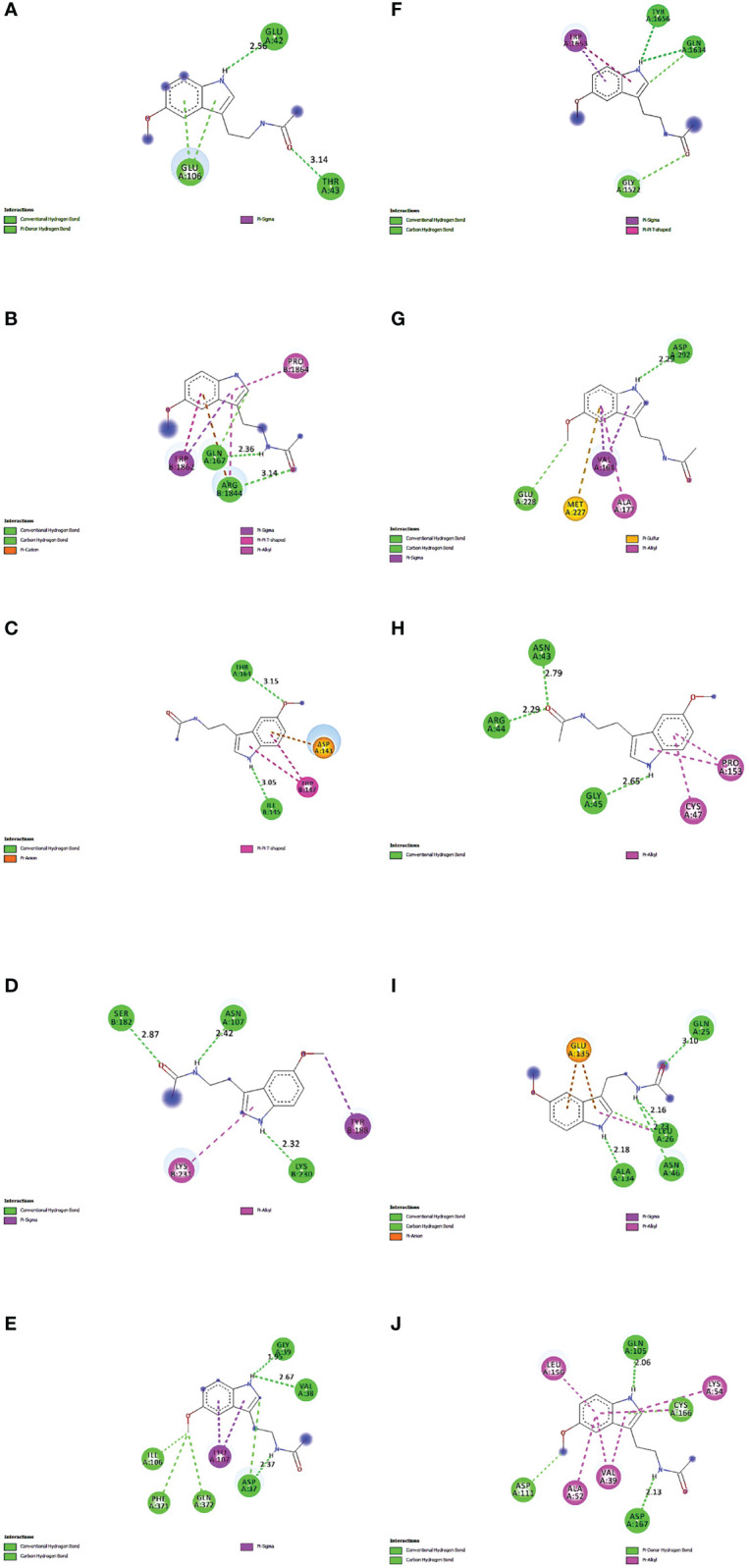
Molecular Docking results of melatonin with top 10 target proteins. **(A)** Binding of melatonin with IL-6. **(B)** Binding of melatonin with TP53. **(C)** Binding of melatonin with ERBB2. **(D)** Binding of melatonin with IL1B. **(E)** Binding of melatonin with MAPK3. **(F)** Binding of melatonin with MTOR. **(G)** Binding of melatonin with AKT1. **(H)** Binding of melatonin with PTGS2. **(I)** Binding of melatonin with TNF. **(J)** Binding of melatonin with MAPK1.

The proteins that were strongly bound to melatonin might alleviate the LR-induced obesity complications. **Figure 9** depicts the binding of core targets to melatonin that possess a strong binding affinity (**Table 2**).

**Table 2:** Top 10 hub protein target list with docking results.

MCC Rank	Name	PDB ID	Target Name	Affinity (kcal/mol)
1	TP53	1GZH	Cellular tumor antigen p53	-5.7
2	AKT1	3MV5	RAC-alpha serine/threonine-protein kinase	-7.4
3	MAPK3	2ZOQ	Mitogen-activated protein kinase 3	-6.4
4	PTGS2	5IKT	Prostaglandin G/H synthase 2	-7.4
5	TNF	5M2J	Tumor necrosis factor	-6.0
6	IL6	1ALU	Interleukin-6	-4.4
7	MAPK1	5NHV	Mitogen-activated protein kinase 1	-6.7
8	ERBB2	1S78	Receptor tyrosine-protein kinase erbB-2	-7.3
9	IL1B	2KH2	Interleukin-1 beta	-6.6
10	MTOR	3JBZ	Serine/threonine-protein kinase mTOR	-6.2

The top 10 hub proteins with its MCC ranking and their binding affinity with melatonin.

## Discussion

Maintaining proper body weight and BMI is essential for everyday living with normal immune function. It is necessary for preventing type 2 diabetes, cardiovascular diseases, arthritis, including some cancers (43). Currently, the incidence of obesity and its related diseases have increased worldwide, including childhood obesity (44). The anti-obesity effect of melatonin and sleep has been proved, but the exact therapeutic targets of leptin resistance-induced obesity are yet to be studied. Hence, for the first time, we used the in-silico network pharmacological analysis to identify the mechanism of action of melatonin against LR-induced Obesity. This will become the preliminary data for reducing LR-induced Obesity by melatonin for further clinical trials. We found ten genes (TP53, AKT1, MAPK3, PTGS2, TNF, IL6, MAPK1, ERBB2, IL1B, MTOR) that might play an important role in reducing LR-induced Obesity (**Table 1**). Molecular docking results also revealed that the hub target genes bound to melatonin with high affinity, particularly AKT1, PTGS2, and ERBB2, can be used to treat LR-induced obesity by melatonin (**Figures 9A-J** and **Table 2**).

### Details of Top 10 Melatonin Targets in Treating Obesity

TP53 is the “guardian of the genome” and a major tumor suppressor gene, and in many types of cancer, major somatic mutations occur in TP53. The significance of p53 in heart disease, obesity, T2DM etc. has been demonstrated (45). Depending on the type of adipocytes, the roles of p53 on adipogenesis differ. Regulation by P53 has a negative impact on the differentiation of brown adipocytes to white adipocytes in the *in vitro* study. TP53 reduces diet-induced and gene related obesity in mouse models and homo sapiens (46). Supplementation of melatonin protects liver function during obesity and diabetes (47). Melatonin reduces the severity of non-alcoholic fatty liver disease (NAFLD). The TP53/p53 gene plays a vital role in regulating glycogen and lipid metabolism. The studies associated with genome disclosed that TP53 plays an essential role in obesity and type 2 diabetes (48).

AKT1, otherwise known as RAC-alpha serine/threonine-protein kinase. *In vivo* studies depicted AKT signaling is involved in brown adipose tissue development. It may regulate adipocyte cell size through pathways like TAG synthesis, lipid uptake, lipolysis, and thermogenesis. Faria et al. (49) demonstrated that melatonin acts locally in the hypothalamus through a mechanism dependent on MT1/MT2 receptors; and activates the phosphatidylinositol 3-kinase/insulin-stimulated RAC-α serine/threonine-protein kinase (PI3K/AKT) pathway. Melatonin stimulates the AKT phosphorylation in the hypothalamus (45% higher than in control, P < 0.05). Melatonin increases glycogen synthesis in mouse liver and reduces glucose production by activating GSK3B *via* insulin-stimulated phosphatidylinositol 3-kinase (PI3K)–AKT signaling (50).

The MTOR (mechanistic target of rapamycin) acts as a key regulator of metabolism, including energy homeostasis and the nutritional status of the cell. Altered signaling from growth factors, cytokines, and hormones are through MTOR and are involved in obesity and insulin resistance (51). The MTOR signaling is involved in adipogenesis, and the treatment of rapamycin inhibited both the proliferation and differentiation of human adipocytes (52). Melatonin treatment concomitantly reduced MTOR phosphorylation in the cells treated with H_2_O_2_ (53).

MAPK1 is known as ERK2, and MAPK3 is known as ERK1, and these are the 2 MAPKs that play an essential role in a wide variety of cellular processes. MAPK1 up-regulates the expression of vital regulators involved in adipogenesis such as CCAAT-enhancer-binding proteins a, b, and d, and peroxisome proliferator-activated receptor g (PPARg) at the beginning stage of adipogenesis (54, 55). Deregulation of the MAPK pathway was observed during obesity and is involved in insulin resistance (56). Melatonin is tightly linked to the PKA and ERK1/2 pathways. Melatonin inhibits proliferation and promotes differentiation of porcine intramuscular preadipocytes. It promotes lipid degradation in intramuscular adipocytes by activating ERK1/2 and PKA (57). Melatonin increases the expression of solute carrier family 39-member 1, activates MAPK/ERK pathways, increases phosphorylation of ERK at 1/2/5 levels and notably inhibits ROS production; and increases the uptake of zinc (58).

The cytokines involved in systemic inflammation and acute-phase reactions are TNFα and IL-6. Elevated serum cytokine levels, particularly IL-6 and TNFα, were observed in obese subjects (59, 60). The obesity patients with increased BMI had abnormal circulation of inflammatory cytokines and are strongly associated in developing severe respiratory failure in COVID-19 (61, 62). Leptin is a cytokine belonging to the family of proinflammatory cytokines and structurally similar to IL-6. Production of leptin can be induced by other inflammatory mediators like TNFα (63). Adipocytes secrete both IL-6 and TNF-α, and their concentration correlates with the percentage and distribution of fat tissue in the body (64). Obesity and liver steatosis are known as low-grade inflammation. In a study, melatonin treatment reduced inflammatory cytokines (TNFα and IL-6) levels in young Zucker diabetic fatty rats (65).

In addition, obesity causes a pro-inflammatory state with the release of several mediators like TNF- α, IL-6, and IL-1β, promoting tumor growth (66). Obesity and type 2 diabetes are predominantly related to non-alcoholic fatty liver disease, including accumulation of hepatic triglyceride and increased pro-inflammatory cytokine expression such as IL-1β (67). A study showed that eight weeks of melatonin supplementation for type 2 diabetes patients significantly reduced the levels of malondialdehyde and IL-1β compared to the control group (68).

Mitochondrial dysfunction is the major factor for developing various disorders by increased production of free radical and nitric oxide. It is reported that the maintenance of mitochondrial homeostasis in obesity and stomach ulcers can be regulated by melatonin treatment (69). The gut dysbiosis increases the ceramide and lipopolysaccharide (LPS) levels in the circulation which activates the inflammatory cytokines like TNFα, IL-6, IL-1β and PTGS2 by nitric oxide synthase (iNOS) and superoxide $\left({\text{O}}_{2}^{-}\right)$ in microglia. 14-3-3 protein is essential in maintaining mitochondrial function (70) *via* modulating butyrate levels. The ceramides negatively regulate 14-3-3 and perturbs normal mitochondrial physiology. Mitochondrial dysfunction plays a major role in gut permeability. Melatonin including butyrate and orexin can positively regulate mitochondrial function including reduction of iNOS and ${\text{O}}_{2}^{-}$ (71).

Increased expression in mRNA levels with specific markers, e.g., prostaglandin-endoperoxide synthase 2 (PTGS2), have been found in HFD-induced Obesity (72). It is reported that the treatment of melatonin significantly reduces the mRNA expression of PTGS2 (73).

The transmembrane receptor ERBB2 is a tyrosine kinase and belongs to the family of EGFR (74) and plays a prominent role in breast cancer. Low levels of ERBB2 are linked to mitochondrial dysfunction, stress response, particularly in cardiac function, and metabolic reprogramming (75). The altered mRNA levels of cytokines like IL-6, TNFα, leptin, adiponectin, C-reactive protein (CRP), and tumor markers like TP53 and ERBB2 in peripheral blood are associated with breast cancer and obesity (60). Overexpressed ERBB2 is the primary therapeutic target for cancer. Many reports explained the anticancer properties of melatonin in ERBB2, particularly in breast cancer (76).

### The Pathways and Functional Modules of Therapeutic Targets of Melatonin Against LR-Induced Obesity

The etiology and pathways involved in obesity, particularly in leptin resistance-induced obesity, is yet to be elucidated. This study revealed the top 33 melatonin and LR-induced obesity targets are involved in the major biological processes in the cells; such as the cellular response to chemical stress, impact of external stimuli, role of autophagy, negative impact of phosphate metabolic process, and regulation of small molecule metabolic process. From the KEGG pathway enrichment analysis (**Figures 7A, B**), the two major pathways 1. Lipid and atherosclerosis (hsa05417) and 2. Endocrine resistance (hsa01522) is enriched with the targets, including the top 10 hub genes (**Table 1**). Obesity and atherosclerosis have similar pathophysiological characteristics and the lipids critically contribute to them; that trigger inflammation and initiation of these diseases is also due to oxidized LDL and free fatty acids (77). Several endocrine alterations are identified and associated with obesity and are reversible by weight loss in endocrine resistance. However, specific endocrine syndromes result in irreversible obesity, like the thyroid hormone levels altered through the hypothalamic-pituitary-thyroid axis. The incidence of hypogonadism and growth hormone deficiencies are associated with abdominal obesity (78).

Further, we analyzed the top 10 KEGG enriched pathways by creating functional modules using a community cluster algorithm. We found that two modules are mainly related to melatonin and obesity and are densely packed (**Figure 6C**). Module 1 consists of the Immune system, Cardiovascular disease, Infectious disease: parasitic, Endocrine and metabolic disease, and Infectious disease: bacterial. Module 2 includes Drug resistance: antineoplastic, Signal transduction, Cancer: specific types, Infectious disease: parasitic, cancer: specific types. The community analysis revealed that melatonin could reduce the risk factor of LR-induced obesity by a wide variety of pathways.

### Biological Processes and Pathway Classes of Melatonin Target LR-Induced Obesity

The pathway class analysis found six classes to be important in reducing the risk of LR-induced obesity by melatonin. We constructed six sub-networks of pathway classes (**Figure 8B**), including nodes from the common top 33 targets, and an increased number of hub genes were involved in each pathway class. MTOR, AKT1, MAPK1 & 3, and TNF genes are present in many networks.

#### Signal Transduction

Melatonin can downregulate the pathways associated with obesity in signal transduction. The mTORC2 can uniquely phosphorylate the kinases like AKT, thereby regulating glucose and lipid metabolism. AKT1 is involved in the browning of white adipocytes. The mTORC2 phosphorylates the AKT1 (hydrophobic motif site HM; S473) and activates AKT1 (79). Administration of melatonin (10 mg/kg) daily for twelve weeks decreased the serum alanine transaminase in hepatic steatosis. Activation of MAPK, JNK, and p38 signaling pathways, pro-inflammatory cytokine expression, including IL-6 and TNFα, are observed in HFD fed mice. The signaling pathways linked with cytokine production, inflammation, and apoptosis are inhibited by melatonin (80).

#### Environmental Adaptation

Exposure to cold induces adaptive thermogenesis in skeletal muscle and brown adipose tissue. The cell responds both externally and internally to these changes (81). The external stimuli such as a change in light intensity, temperature, humidity, etc., while an internal stimulation is a change within the cell itself. In liver and adipose tissue, with obesity, molecular markers of stress and autophagy were increased (82). Melatonin regulates several signaling pathways, including the ERK and MAPK pathways which regulate the immune system. In addition, it can reduce H_2_O_2_-induced oxidative stress by altering the ERK/AKT/NFkB pathways (83).

#### Endocrine System & Endocrine and Metabolic Disease

Melatonin regulates different physiological and neuroendocrine functions in mammals. The role of melatonin in obesity and T2DM is widely studied. The activation of kinases (PI3K, AKT, and ERK1/2) by melatonin is potentially relevant in regulating glucose homeostasis (44).

#### Cardiovascular Disease

Studies have demonstrated that melatonin has significant effects on hypertension, valvular heart diseases, vascular diseases, and lipid metabolism. Melatonin can be a therapeutic option for treating cardiovascular disease. Melatonin inhibits the propagation of smooth myocytes in the pulmonary arteries and decreases the MAPK1/3 (ERK1/2) expression and phosphorylation of AKT (20).

#### Drug Resistance

Intervention of melatonin in the dominant signal transduction pathways, such as AKT and MAPK, is not dependent on its antioxidant properties. The pleiotropic effects of melatonin can modulate the drug targets expression and phosphorylation status, increasing the sensitization to antineoplastic agents (84, 85).

### Conclusions

To conclude, melatonin treatment could alleviate the complications of LR-induced obesity by regulating the top 10 targets (TP53, AKT1, MAPK3, PTGS2, TNF, IL6, MAPK1, ERBB2, IL1B, MTOR). These improve the biological processes (cellular response to chemical stress, external stimuli, autophagy, negative impact in phosphate metabolism, regulation of small molecule metabolism and signaling pathways such as (lipid and atherosclerosis and endocrine resistance). In addition, melatonin can improve LR-induced obesity by modulating various pathway classes, signal transduction, endocrine system, endocrine and metabolic disease, environmental adaptation, drug resistance antineoplastic, and cardiovascular diseases.

## Data Availability Statement

The original contributions presented in the study are included in the article/**Supplementary Material**. Further inquiries can be directed to the corresponding author.

## Author Contributions

Vision of the manuscript and editing including revisions made by VN. VS collected literature, made results and wrote the manuscript. All authors contributed to the article and approved the submitted version.

## Funding

This work is funded by the UGC-BSR Faculty fellowship.

## Conflict of Interest

The authors declare that the research was conducted in the absence of any commercial or financial relationships that could be construed as a potential conflict of interest.

## Correction Note

A correction has been made to this article. Details can be found at: 10.3389/fendo.2025.1670260.

## Publisher’s Note

All claims expressed in this article are solely those of the authors and do not necessarily represent those of their affiliated organizations, or those of the publisher, the editors and the reviewers. Any product that may be evaluated in this article, or claim that may be made by its manufacturer, is not guaranteed or endorsed by the publisher.

## Abbreviations

AKT, Protein kinase B; BBB- Blood-brain barrier; BMI, Body mass index; COVID-19, Coronavirus disease 2019; EGFR, Epidermal growth factor receptor; ERBB2, erb-b2 receptor tyrosine kinase 2; GO, Gene Ontology; HFD, High fat diet; IL6, Interleukin 6; IL1B, Interleukin 1 beta; KEGG, Kyoto Encyclopedia of Genes and Genomes; LDL, Low-density lipoprotein; LR, Leptin Resistance; MAPK, Mitogen-activated protein kinase; MTOR, mechanistic target of rapamycin; PDB, Protein Data Bank; PPI, Protein-protein interaction; PTGS2, prostaglandin-endoperoxide synthase 2; T2DM, Type 2 Diabetes Mellitus; TP53, Tumor protein P53; TNF, Tumor necrosis factor; WHO, World Health Organization.
