# Correction: Therapeutic target analysis and molecular mechanism of melatonin-treated leptin resistance induced obesity: a systematic study of network pharmacology

**DOI:** 10.3389/fendo.2025.1670260

**Published:** 2025-08-15

**Authors:** Vennila Suriyagandhi, Vasanthi Nachiappan

**Affiliations:** Biomembrane Lab, Department of Biochemistry, School of Life Sciences, Bharathidasan University, Tamilnadu, India

**Keywords:** melatonin, obesity, leptin resistance, bioinformatic analysis, network topological analysis

Author “Vennila Suriyagandhi” was erroneously spelled as “Vennila Suriagandhi”.

The original version of this article has been updated.

