# Supplementary material for: Therapeutic Target Analysis and Molecular Mechanism of Melatonin - Treated Leptin Resistance Induced Obesity: A Systematic Study of Network Pharmacology
Source: Front Endocrinol (Lausanne). 2022 Jul 22;13:927576. doi: 10.3389/fendo.2022.927576 (PMC9352999; doi:10.3389/fendo.2022.927576)
Supplement: Supplementary file 1 [file DataSheet_1.docx]

**Supplementary figure 1:** Common genes of obesity and leptin resistance


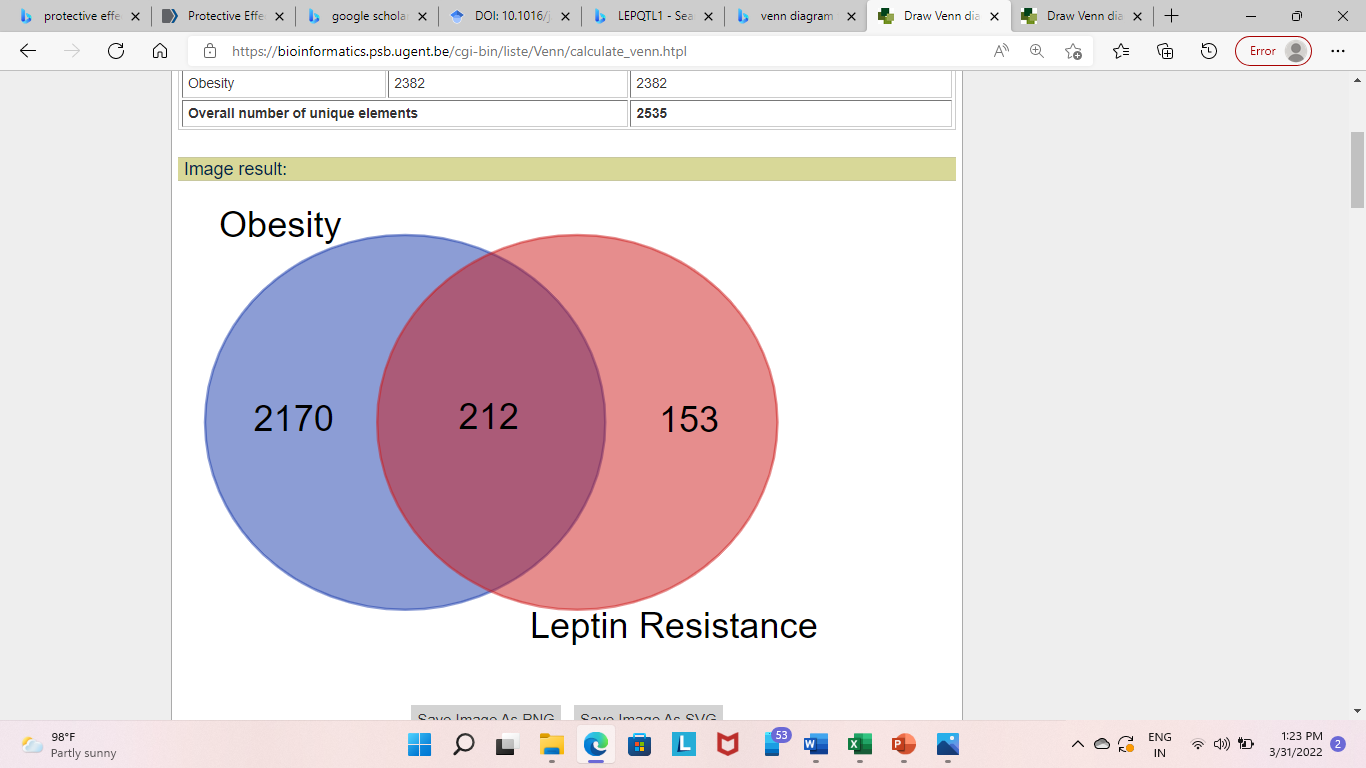


**Supplementary figure 1:** Venn diagram of Obesity and Leptin resistance. We have found 2382 genes for obesity and 365 genes for leptin resistance. From that common 212 intersecting genes of LR and obesity obtained by Venn diagram analysis.
